# Editorial: The proceedings of mitochondria apoptosis and cancer (MAC 2021) virtual symposium

**DOI:** 10.3389/fcell.2022.1118314

**Published:** 2023-01-04

**Authors:** Shazib Pervaiz

**Affiliations:** ^1^ NUS Centre for Cancer Research (N2CR), Yong Loo Lin School of Medicine, National University of Singapore, Singapore, Singapore; ^2^ Department of Physiology, Yong Loo Lin School of Medicine, National University of Singapore, Singapore, Singapore; ^3^ National University Cancer Institute, National University Health System, Singapore, Singapore

**Keywords:** mitochondria, MOMP, cancer, sirt3, MCS

## Introduction

Mitochondria Apoptosis and Cancer (MAC) was first organized and held as an EMBO workshop in Prague, CZ, in 2009 (MAC′09). Following the enthusiastic response and success of the first meeting, the scientific committee decided to make MAC symposium a regular biannual event. MAC 2021 was the seventh symposium in the series, organized and hosted by Department of Physiology, Yong Loo Lin School of Medicine, National University of Singapore, Singapore, as a virtual event due to the COVID-19 pandemic. The virtual symposium provided ample opportunities for discussion and exchange of ideas amongst the speakers and participants. As with the previous MAC symposia, opinion leaders as well as young and emerging researchers touched on the multi-faceted roles of mitochondrial metabolism in cell fate signaling during health and various pathologies, such as mitochondrial adaptation during hypoxia, the important role of OPA1 in maintaining cristae structure and mitochondrial fission, post-translational modifications of proteins involved in preventing mitochondrial permeability, mitochondrial transfer as a novel cancer promoting mechanism and targeting mitochondrial metabolism to overcome drug resistance in mutant KRAS addicted cancers.

## Mitochondrion: A multifaceted organelle

In addition to their critical role in processes and pathways maintaining cellular homeostasis, mitochondria also function as a central hub controlling various execution signals. To that end, mitochondrial outer membrane permeabilization (MOMP) induced upon translocation and oligomerization of pro-apoptotic proteins of the Bcl-2 family, such as Bax, Bak and Bok, facilitate the egress of cytochrome C and apoptosis inducing factor (AIF) that amplify apoptotic signaling (Wei et al., 2001). MOMP is regulated by the interplay between BH3-only proteins, Bim, Bad, Noxa and Puma, and anti-apoptotic members of the Bc-2 family, in particular Bcl-2, Bcl-xL and Mcl-1 (Chen et al., 2015). MOMP and its regulation have also been linked to cellular redox state, such as observations linking ROS to Bax translocation which in turn further amplifies mitochondrial ROS to compromise mitochondrial outer membrane integrity (Hirpara et al., 2000; Ahmad et al., 2004; Garcia-Perez et al., 2012). Interestingly, a non-canonical redox-dependent mechanism involving mitochondrial metabolism has also been demonstrated for the anti-apoptotic activity of Bcl-2, which involves it´s post-translational modification (Clement et al., 2003; Chen and Pervaiz, 2007; Chen and Pervaiz, 2010; Low et al., 2014; Chong et al., 2020). Furthermore, mitochondria have been shown to play a critical role in non-apoptotic cell death, such as *via* redox-mediated execution of necroptosis and ferroptosis (Stockwell et al., 2017) as well as pathogen-induced inflammasome-mediated pyroptosis. Recent evidence also implicates MOMP in inflammation, thus indicating a non-lethal role. In this context, inner membrane remodelling or rupture allows for the release of mitochondrial DNA, thus triggering the cGAS-STING pathways to promote inflammation (McArthur et al., 2018). This Research Topic highlights emerging roles of mitochondria and mitochondrial metabolism in the various processes and pathways regulating health and disease (elegantly reviewed in (Bock and Tait, 2020).

Sassano et al. provide a review of membrane contact sites (MCS) and their role in the exchange of ions and other substances across organelles, particularly focusing on endoplasmic reticulum (ER)-Mitochondria contact sites (EMCS). MCS are highly dynamic molecular bridges involved in structural integrity and inter-organellar communication *via* lipid-mediated signaling (Balla et al., 2019). Dysregulation of MCS is associated with neurodegenerative disorders, lysosomal storage disease and cancer. The review specifically focuses on two important functional attributes of EMCS, Ca^2+^ fluxes and lipid transfer, between ER and mitochondria and how these functions are deregulated in cancer. The crosstalk between ER and mitochondria, including the role of various interacting proteins (transporters), in Ca^2+^ signaling and its dysregulation in cancer has been well documented (Cardenas et al., 2016), however, the mechanism by which EMCS regulate mitochondrial lipid homeostasis remains less well characterized. EMCS are involved in the synthesis of phosphatidylserine (PS) and its conversion to phosphatidylethanolamine (PE), and cardiolipin, which is synthesized in the mitochondria, however, majority is transported from the ER *via* the MCS (Vance, 2014). Disruption of lipid homeostasis has implications for pathologies involving deregulated autophagy, apoptosis, ferroptosis and necroptosis as membrane damage promotes MOMP as well as mitochondrial dynamics *via* remodelling inner membrane and cristae integrity.

Lai et al. provide a comprehensive account of the role that mitochondria play in various cell death modalities in osteosarcoma (OS), a highly aggressive and most common primary bone cancer affecting children and young adults with high metastatic potential and limited treatment options. Chemotherapy following surgical resection remains the main therapeutic option, however, development of chemoresistance warrants the need for a better understanding of disease mechanism(s) and identification of biomarkers as well as novel drug targets. The review highlights the involvement of various regulated cell death pathways and their deregulation in OS, including the role of apoptosis inhibitors such as Bcl-2 family and potential use of BH3 mimetic Bcl-2 inhibitors, the role of ROS modulation that impacts ferroptosis and other modalities such as necroptosis and pyroptosis, the prognostic value of autophagic markers and use of autophagy inhibitors, and the largely untapped area of mitochondrial metabolism. It is tempting to speculate that regulating intracellular ROS might be a potential therapeutic strategy against OS, considering how cellular redox metabolism impacts most cell death modalities. To that end, redox dependent non-canonical apoptosis regulatory activity of Bcl-2 as well as pro-survival signaling by other oncoproteins have been recently demonstrated (Hirpara et al., 2020; Raman et al., 2020; Yee et al., 2021).

The multi-faceted role of mitochondrial NAD^+^ deacetylase, Sirtuin 3 (SIRT3), in various cell death modalities is reviewed by Yapryntseva et al. This review discusses the role of SIRT3 in regulating MOMP and various cell death modalities by deacetylation-mediated activation of transcription factors, such as FOXO3a and Nrf2, which control the expression of anti-oxidant genes, or *via* deacetylating mitochondrial anti-oxidant proteins such as succinate dehydrogenase (SDH) and proteins of the electron transport chain. This anti-oxidant effect results in suppression of Ras mediated PI3K/Akt and MAPK activation, thus suppressing carcinogenesis (Torrens-Mas et al., 2017), however, by maintaining a low oxidant environment SIRT3 can also have the opposite effect of promoting oncogenesis (Kim et al., 2020). This is also corroborated by the ability of SIRT3 to inhibit iron uptake through the transferrin receptor (TFR1), which could blunt the effect of drugs that use ferroptosis for death execution (Jeong et al., 2015). Also, by activating autophagy/mitophagy, SIRT3 can also inhibit inflammasome assembly thus regulating pyroptosis (Liu et al., 2018). The divergent effects of SIRT3 on cell fate are in line with recent reports indicating the dichotomy of redox signaling in cancer cell fate decisions. Whether the divergent effects of SIRT3 is a function of distinct redox milieu remains to be elucidated.

Hsu et al. provide a focused review on the DNA anchor protein of the YAP transcription complex, TEAD4 (TEA Domain Transcription Factor 4), which plays an essential role in cell growth, differentiation, and proliferation. Conventionally, TEAD4 requires YAP/TAZ for its transcriptional activity; however, a YAP-independent function has also been described. Notably, increased expression of TEAD4 has been described in a variety of human cancers, which could be linked to its unique ability to transcribe mitochondrial and nuclear-encoded OXPHOS genes, thereby keeping cellular ROS levels at a manageable level (Kumar et al., 2018). A link between viral infections such as hepatitis virus and sendai virus and TEAD4 activity has also been demonstrated, which might explain viral-induced carcinogenesis in the gastrointestinal tract (Jiao et al., 2018). Notably, TEAD4 knockout mice are viable after embryo implantation stage and with functional redundancy with other TEAD members, specific targeting of TEAD4-mediated signaling in cancer cells could have potential implications. Along these lines, the authors discuss the difficulty in directly targeting TEAD4 due to its pleiotropic function, but present evidence to support the use of strategies to disrupt YAP/TEAD4 interaction for the preferential execution of cancer cells.

Nahacha et al. summarize findings on mitochondria transport proteins Miro and their role in mitochondrial transfer in cancer cells. This is relevant considering that cancer cells with damaged OXPHOS machinery are able to import mitochondria from healthy neighboring cells for their sustenance, thus corroborating the role of mitochondrial metabolism in cancer cell survival and progression. The spatial localization and transport of mitochondria are mediated through the function of a family of small GTPases, Miro, localized in the outer membrane. These proteins are also involved in ER-Mitochondria contact sites by contributing to the MICOS complex and thereby regulating Ca^2+^ metabolism and mitophagy (Saotome et al., 2008). The authors focus on the important role of Miro proteins in intra- and intercellular transport and trafficking of mitochondria and its functional significance in various processes associated with carcinogenesis such as migration and metastasis.

## Unresolved questions

While the role of cellular redox metabolism appears to play an important role in the various pathways and processes involving the mitochondria, there are several interesting questions that remain unanswered, such as: Is MOMP induction a point of no return? What are the molecular mechanisms underlying the divergent signaling by mitochondrial ROS in cell fate decisions? What cell states distinguish the tumor suppressor function of SIRT3 from its cancer promoting activity? Do mitochondria function as central hub connecting various cell death pathways? Addressing these important questions will unravel novel mechanisms and targets with therapeutic implications against a host of human pathologies involving mitochondrial metabolism.

## References

[B1] Ahmad K. A. Iskandar K. B. Hirpara J. L. Clement M. V. Pervaiz S. (2004). Hydrogen peroxide-mediated cytosolic acidification is a signal for mitochondrial translocation of Bax during drug-induced apoptosis of tumor cells. Cancer Res. 64, 7867–7878. 10.1158/0008-5472.CAN-04-0648 15520193

[B2] Balla T. Kim Y. J. Alvarez-Prats A. Pemberton J. (2019). Lipid dynamics at contact sites between the endoplasmic reticulum and other organelles. Annu. Rev. Cell Dev. Biol. 35, 85–109. 10.1146/annurev-cellbio-100818-125251 31590585

[B3] Bock F. J. Tait S. W. G. (2020). Mitochondria as multifaceted regulators of cell death. Nat. Rev. Mol. Cell Biol. 21, 85–100. 10.1038/s41580-019-0173-8 31636403

[B4] Cardenas C. Muller M. McNeal A. Lovy A. Jana F. Bustos G. (2016). Selective vulnerability of cancer cells by inhibition of Ca(2+) transfer from endoplasmic reticulum to mitochondria. Cell Rep. 15, 219–220. 10.1016/j.celrep.2016.03.045 27050774

[B5] Chen H. C. Kanai M. Inoue-Yamauchi A. Tu H. C. Huang Y. Ren D. (2015). An interconnected hierarchical model of cell death regulation by the BCL-2 family. Nat. Cell Biol. 17, 1270–1281. 10.1038/ncb3236 26344567 PMC4589531

[B6] Chen Z. X. Pervaiz S. (2007). Bcl-2 induces pro-oxidant state by engaging mitochondrial respiration in tumor cells. Cell Death Differ. 14, 1617–1627. 10.1038/sj.cdd.4402165 17510660

[B7] Chen Z. X. Pervaiz S. (2010). Involvement of cytochrome c oxidase subunits Va and Vb in the regulation of cancer cell metabolism by Bcl-2. Cell Death Differ. 17, 408–420. 10.1038/cdd.2009.132 19834492

[B8] Chong S. J. F. Iskandar K. Lai J. X. H. Qu J. Raman D. Valentin R. (2020). Serine-70 phosphorylated Bcl-2 prevents oxidative stress-induced DNA damage by modulating the mitochondrial redox metabolism. Nucleic Acids Res. 48, 12727–12745. 10.1093/nar/gkaa1110 33245769 PMC7736805

[B9] Clement M. V. Hirpara J. L. Pervaiz S. (2003). Decrease in intracellular superoxide sensitizes Bcl-2-overexpressing tumor cells to receptor and drug-induced apoptosis independent of the mitochondria. Cell Death Differ. 10, 1273–1285. 10.1038/sj.cdd.4401302 12894215

[B10] Garcia-Perez C. Roy S. S. Naghdi S. Lin X. Davies E. Hajnoczky G. (2012). Bid-induced mitochondrial membrane permeabilization waves propagated by local reactive oxygen species (ROS) signaling. Proc. Natl. Acad. Sci. U. S. A. 109, 4497–4502. 10.1073/pnas.1118244109 22393005 PMC3311374

[B11] Hirpara J. L. Seyed M. A. Loh K. W. Dong H. Kini R. M. Pervaiz S. (2000). Induction of mitochondrial permeability transition and cytochrome C release in the absence of caspase activation is insufficient for effective apoptosis in human leukemia cells. Blood 95, 1773–1780. 10.1182/blood.v95.5.1773.005k17_1773_1780 10688837

[B12] Hirpara J. L. Subramaniam K. Bellot G. Qu J. Seah S. Loh T. (2020). Superoxide induced inhibition of death receptor signaling is mediated via induced expression of apoptosis inhibitory protein cFLIP. Redox Biol. 30, 101403. 10.1016/j.redox.2019.101403 31954371 PMC6965745

[B13] Jeong S. M. Lee J. Finley L. W. Schmidt P. J. Fleming M. D. Haigis M. C. (2015). SIRT3 regulates cellular iron metabolism and cancer growth by repressing iron regulatory protein 1. Oncogene 34, 2115–2124. 10.1038/onc.2014.124 24909164 PMC4747239

[B14] Jiao S. Guan J. Chen M. Wang W. Li C. Wang Y. (2018). Targeting IRF3 as a YAP agonist therapy against gastric cancer. J. Exp. Med. 215, 699–718. 10.1084/jem.20171116 29339449 PMC5789414

[B15] Kim Y. S. Gupta Vallur P. Jones V. M. Worley B. L. Shimko S. Shin D. H. (2020). Context-dependent activation of SIRT3 is necessary for anchorage-independent survival and metastasis of ovarian cancer cells. Oncogene 39, 1619–1633. 10.1038/s41388-019-1097-7 31723239 PMC7036012

[B16] Kumar R. P. Ray S. Home P. Saha B. Bhattacharya B. Wilkins H. M. (2018). Regulation of energy metabolism during early mammalian development: TEAD4 controls mitochondrial transcription. Development 145, dev162644. 10.1242/dev.162644 30201685 PMC6198476

[B17] Liu P. Huang G. Wei T. Gao J. Huang C. Sun M. (2018). Sirtuin 3-induced macrophage autophagy in regulating NLRP3 inflammasome activation. Biochim. Biophys. Acta Mol. Basis Dis. 1864, 764–777. 10.1016/j.bbadis.2017.12.027 29277324

[B18] Low I. C. Loh T. Huang Y. Virshup D. M. Pervaiz S. (2014). Ser70 phosphorylation of Bcl-2 by selective tyrosine nitration of PP2A-B56δ stabilizes its antiapoptotic activity. Blood 124, 2223–2234. 10.1182/blood-2014-03-563296 25082878

[B19] McArthur K. Whitehead L. W. Heddleston J. M. Li L. Padman B. S. Oorschot V. (2018). BAK/BAX macropores facilitate mitochondrial herniation and mtDNA efflux during apoptosis. Science 359, eaao6047. 10.1126/science.aao6047 29472455

[B20] Raman D. Chong S. J. F. Iskandar K. Hirpara J. L. Pervaiz S. (2020). Peroxynitrite promotes serine-62 phosphorylation-dependent stabilization of the oncoprotein c-Myc. Redox Biol. 34, 101587. 10.1016/j.redox.2020.101587 32512497 PMC7280771

[B21] Saotome M. Safiulina D. Szabadkai G. Das S. Fransson A. Aspenstrom P. (2008). Bidirectional Ca2+-dependent control of mitochondrial dynamics by the Miro GTPase. Proc. Natl. Acad. Sci. U. S. A. 105, 20728–20733. 10.1073/pnas.0808953105 19098100 PMC2634948

[B22] Stockwell B. R. Friedmann Angeli J. P. Bayir H. Bush A. I. Conrad M. Dixon S. J. (2017). Ferroptosis: A regulated cell death nexus linking metabolism, redox Biology, and disease. Cell 171, 273–285. 10.1016/j.cell.2017.09.021 28985560 PMC5685180

[B23] Torrens-Mas M. Pons D. G. Sastre-Serra J. Oliver J. Roca P. (2017). SIRT3 silencing sensitizes breast cancer cells to cytotoxic treatments through an increment in ROS production. J. Cell Biochem. 118, 397–406. 10.1002/jcb.25653 27420645

[B24] Vance J. E. (2014). MAM (mitochondria-associated membranes) in mammalian cells: Lipids and beyond. Biochim. Biophys. Acta 1841, 595–609. 10.1016/j.bbalip.2013.11.014 24316057

[B25] Wei M. C. Zong W. X. Cheng E. H. Lindsten T. Panoutsakopoulou V. Ross A. J. (2001). Proapoptotic BAX and BAK: A requisite gateway to mitochondrial dysfunction and death. Science 292, 727–730. 10.1126/science.1059108 11326099 PMC3049805

[B26] Yee Y. H. Chong S. J. F. Kong L. R. Goh B. C. Pervaiz S. (2021). Sustained IKKβ phosphorylation and NF-κB activation by superoxide-induced peroxynitrite-mediated nitrotyrosine modification of B56γ3 and PP2A inactivation. Redox Biol. 41, 101834. 10.1016/j.redox.2020.101834 33838472 PMC8056462

